# Antihypertensive Action of Allantoin in Animals

**DOI:** 10.1155/2014/690135

**Published:** 2014-03-12

**Authors:** Mei-Fen Chen, Jo-Ting Tsai, Li-Jen Chen, Tung-Pi Wu, Jia-Jang Yang, Li-Te Yin, Yu-lin Yang, Tai-An Chiang, Han-Lin Lu, Ming-Chang Wu

**Affiliations:** ^1^Department of Food Science, National Pingtung University of Science and Technology, Neipu, Pingtung City 91201, Taiwan; ^2^College of Medicine and Life Science, Chung Hwa University of Medical Technology, Rende District, Tainan City 71703, Taiwan; ^3^Department of Radiation Oncology, Taipei Medical University-Shuang Ho Hospital, and College of Medicine, Taipei Medical University, Taipei City 10361, Taiwan; ^4^Institute of Basic Medical Sciences, College of Medicine, National Cheng Kung University, Tainan City 70101, Taiwan; ^5^Department of Obs/Gyn, Tainan SinLau Hospital, The Presbyterian Church in Taiwan, Tainan City 70142, Taiwan; ^6^Department of Chinese Medicine, Tainan SinLau Hospital, The Presbyterian Church in Taiwan, Tainan City 70142, Taiwan

## Abstract

The agonists of imidazoline I-1 receptors (I-1R) are widely used to lower blood pressure. It has been indicated that guanidinium derivatives show an ability to activate imidazoline receptors. Also, allantoin has a chemical stricture similar to guanidinium derivatives. Thus, it is of special interest to characterize the effect of allantoin on I-1R. In conscious male spontaneous hypertensive rats (SHRs), mean blood pressure (MBP) was recorded using the tail-cuff method. Furthermore, the hemodynamic analyses in catheterized rats were applied to measure the actions of allantoin in vivo. Allantoin decreased blood pressures in SHRs at 30 minutes, as the most effective time. Also, this antihypertensive action was shown in a dose-dependent manner from SHRs treated with allantoin. Moreover, in anesthetized rats, allantoin inhibited cardiac contractility and heart rate as showing in hemodynamic *dP*/*dt* max significantly. Also, the peripheral blood flow was markedly increased by allantoin. Both actions were diminished by efaroxan at the dose sufficient to block I-1R. Thus, we suggest that allantoin, as I-1R agonist, has the potential to develop as a new therapeutic agent for hypertension in the future.

## 1. Introduction

Neurotransmitters after binding to specific receptors are known to involve in the regulation of cardiovascular functions, especially the arterial blood pressure. In this regulation, noradrenaline, acetylcholine, serotonin, angiotensin II, and g-amino-butyric acid are widely introduced as the central regulators of blood pressure [1]. Potentially, most of neurotransmitters and/or receptors in brain could be considered as the targets in development of centrally acting antihypertensive agents [2].

Allantoin is known rich contained in in yam (*Dioscorea spp.*) as the principle active compound [3]. Yam is an important plant that is widely used in drug industry, while* Dioscorea rhizome* contained ureides including allantoin for the prevention of inflammation and ulcers [4]. Actually, the herbs from Dioscoreaceae are introduced to be merit in the improvement of diabetic disorders [5]. In Chinese traditional medicine, Shan-Yaw (*Dioscorea opposita*) is effective to improve insulin resistance [6] and it has also been characterized in animals [7], while allantoin is mentioned to be contained in this herb [8].

The presence of imidazoline receptors in brain seems to be related to the central regulation of blood pressure [9]. It has been dissociated the centrally mediated effects of clonidine (an imidazoline compound) on blood pressure from those of catecholamines [9]. Accordingly, the detailed radioligand binding studies largely confirmed the presence of imidazoline receptors [10]. After the characterization of agmatine as the endogenous ligand of this receptor, 3 subtypes of imidazoline receptors have been proposed; activation of I-1 receptors regulates blood pressure [11], whereas I-3 receptors participate in insulin release [12] and activation of I-2 receptors (I-2R) increases glucose uptake into muscle cells [13, 14]. Moreover, it has been documented that compounds with guanidine-like structures may bind to imidazoline receptors [15], while metformin has been identified to this group [16]. Because allantoin also belongs to guanidinium derivative, it is of special interest to understand the effect of allantoin on I-1R. Thus, we speculated that allantoin may have central antihypertensive activity through activation of I-1R. Then, in the present study, we investigated the antihypertensive action of allantoin in relation to I-1R in both normal rats and spontaneous hypertensive rats (SHRs).

## 2. Material and Methods

### 2.1. Animals

Twelve-week-old male Wistar rats and spontaneously hypertensive rats (SHR), weighing from 250 to 300 g, were obtained from the Animal Center of National Cheng Kung University Medical College. The rats were housed individually in plastic cages under standard laboratory conditions. They were kept under a 12 h light/dark cycle and had free access to food and water. All experiments were performed under anesthesia with 2% isoflurane, and all efforts were made to minimize the animals' suffering. The animal experiments were approved and conducted in accordance with local institutional guidelines for the care and use of laboratory animals, and the experiments conformed to the Guide for the Care and Use of Laboratory Animals as well as the guidelines of the Animal Welfare Act.

### 2.2. Drug Administration

Animals were randomly assigned into four groups: (I) the control group (*n* = 8) treated with the vehicle, saline (0.9% sodium chloride, i.v.); (II) the allantoin group (*n* = 8) treated by intravenous injection of allantoin at 0.5 mg/kg as described previously [17, 18]; (III) the allantoin + efaroxan group (*n* = 8) treated with allantoin at the most effective dose (0.5 mg/kg, i.v.) according to previous report [17, 18] and efaroxan at effective dose (1.5 mg/kg, i.v.) [17] 30 minutes before injection of allantoin; and (IV) the allantoin treated SHRs group (*n* = 8) treated by intravenous injection of allantoin at various dose for desired time. Because allantoin has been documented to be easily degraded in intestinal tract [19] for resulting in a marked loss of activity after oral administration [20, 21], we administered it using intravenous injection in the present study.

### 2.3. Determination of Mean Blood Pressure

After treatment of allantoin, the rats were placed into a holder for the determination of the mean blood pressure (MBP) using a noninvasive tail-cuff monitor (MK2000; Muromachi Kikai, Tokyo, Japan). The values for each animal were determined in triplicate.

### 2.4. Determination of Blood Flow

Then, the rats were anesthetized and cannulated in the right femoral artery with polyethylene catheters (PE-50). Mean arterial pressure (MAP) and heart rate (HR) were recorded using a polygraph (MP35, BIOPAC, Goleta, Calif.). The rat's trachea was intubated for artificial ventilation (Small Animal Ventilator Model 683, Harvard Apparatus, Holliston, Mass.) at 50 breaths/min with a tidal volume of 8 mL/kg and a positive end expiratory pressure of 5 cm H_2_O. After incision into the rat's chest at the third intercostal space to expose the heart, a small section (1 cm long) of the ascending aorta was freed from the connective tissue. A Transonic Flowprobe (2.5PSB923, Transonic System Inc., Ithaca, N.Y.) was implanted around the root of the ascending aorta and connected to a Transonic transit-time blood flowmeter (T403, Transonic System Inc.). The MAP, HR, and blood flow were record for further analysis.

### 2.5. Catheterization for Hemodynamic *dP*/*dt* Measurement

Temporary pacing leads were used for the short-term study and were placed in the right atrium and RV apex. A venogram image in 2 different angulations (left anterior oblique 30° and anteroposterior) was obtained to determine the anatomy of the coronary sinus venous system. An LV pacing electrode (IX-214; iWorx Systems, Inc., Dover, NH, USA) was placed either in the free wall region via the lateral or posterior vein or in the anterior region via the great cardiac vein. After femoral artery and venous puncture using the Seldinger technique [22], pressure transducer catheters were inserted into the heart to provide the RV, aortic, mean blood, and LV pressures. Pressure catheters and pacing leads were connected to an external pacing computer (iWorx Systems, Inc., Dover, NH, USA) to monitor the heart rate and to acquire hemodynamic signals. Body temperature of the rats was also maintained at 37.5°C throughout whole procedure.

### 2.6. Statistical Analysis

Results were expressed as mean ± SE of each group. Statistical analysis was carried out using ANOVA analysis and the Newman-Keuls post hoc analysis. Statistical significance was set as *P* < 0.05.

## 3. Results

### 3.1. Effects of Allantoin on the Blood Pressure in Conscious SHRs

**Figure 1:**
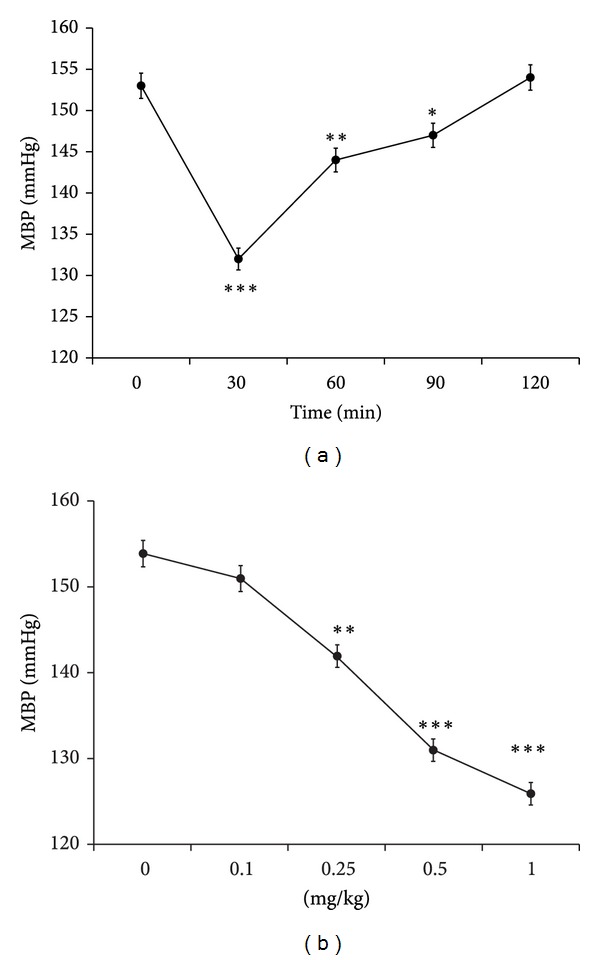
Antihypertensive action of allantoin in spontaneous hypertensive rats (SHRs). Time course (a) and dose-dependent (b) decrease of mean blood pressure (MBP) induced by allantoin in conscious spontaneously hypertensive rats (SHRs). Data represent the mean ± SEM of eight animals (*n* = 8). *P < 0.05, **P < 0.01, and ***P < 0.001 compared with control (at zero).

We investigated the most effective time point of allantoin using the intravenous injection of allantoin into SHRs for 0–120 minutes at the dose of 0.5 mg/kg according to previous study [17]. The most effective time point of 30 min was then obtained and used for further study (Figure 1(a)). Intravenous injection of allantoin from 0.1 to 0.5 mg/kg decreased MBP in conscious SHRs in a dose-dependent manner (Figure 1(b)). Then, the time point of 30 min and the dose of 0.5 mg/kg were used for further experiments.

### 3.2. The Reduction of MAP and HR by Allantoin in Rats Was Diminished by Blockade of I-1R Using Efaroxan

**Figure 2:**
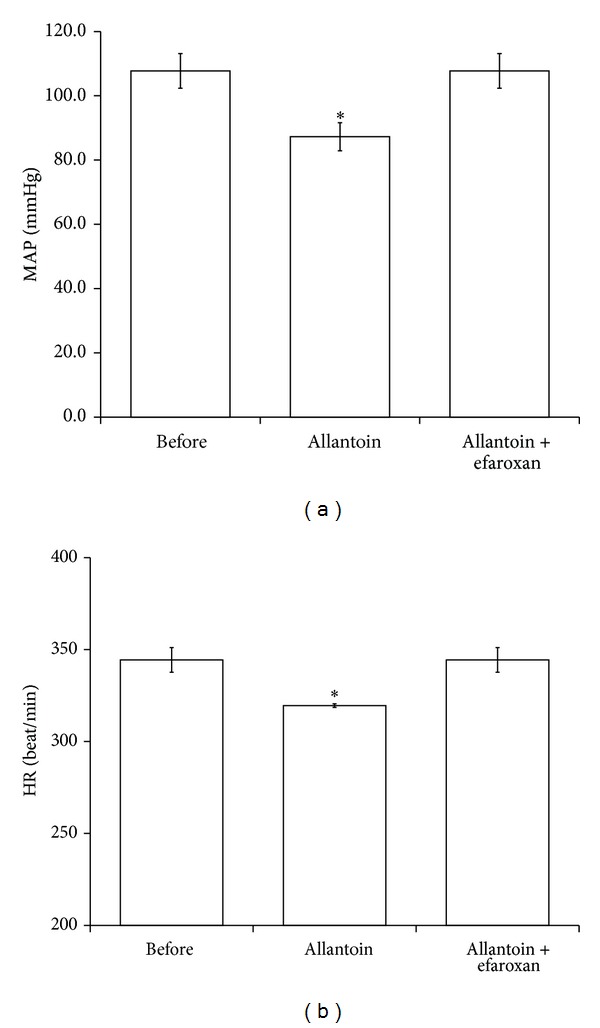
Effects of allantoin on mean arterial pressure (MAP) and heart rate (HR) in rats. The representative picture shows the change in MAP and HR caused by allantoin in anesthetized rats. HR and MAP were recorded in anesthetized rats treated with allantoin or cotreatment with efaroxan. The changes in MAP (a) and HR (b) were recorded at 30 min after injection of allantoin. All values are presented as mean ± SEM (*n* = 8). *P < 0.05 as compared to the data in before.

In order to clarify that allantoin may produce antihypertensive effect through central I-1R, we measured the MAP and HR in normal rats. The MAP and HR were markedly decreased after injection of allantoin (Figure 2). However, as shown in Figure 2, this action was extinguished by I-1R specific antagonist named efaroxan at the effective dose as showed in previous report [17].

### 3.3. Effect of Allantoin on Cardiac Performance in the Anesthetized Rats

**Figure 3:**
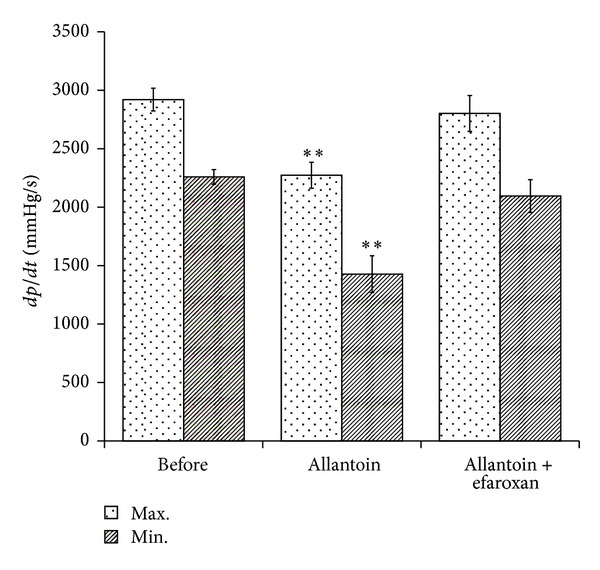
Effects of allantoin on cardiac performance in anesthetized rats. The effects of coadministration of allantoin and/or efaroxan were investigated in the anesthetized rats. The changes in hemodynamic *dP*/*dt* were recorded at 30 min after injection of allantoin. All values are presented as mean ± SEM (*n* = 8). **P < 0.01 as compared to the data in before.

The *dP*/*dt* was significantly reduced by allantoin (0.5 mg/kg, i.v.) after treatment for 30 min in the anesthetized rats, compared with the vehicle-treated control. However, as shown in Figure 3, this effect disappeared by coadministration of efaroxan at effective dose (1.5 mg/kg, i.v.) [17].

### 3.4. Effect of Allantoin on Peripheral Blood Flow in the Anesthetized Rats

**Figure 4:**
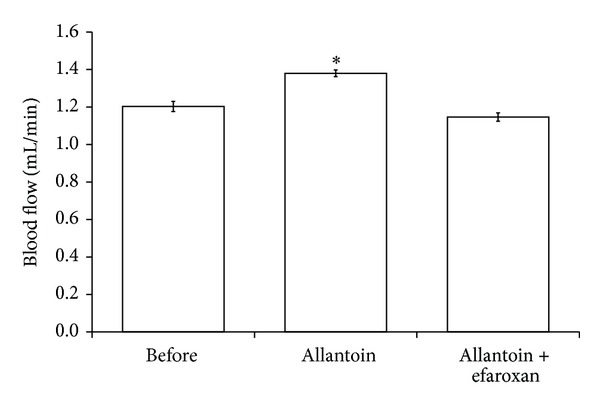
Effects of allantoin on peripheral blood flow in anesthetized rats. The effects of allantoin or cotreatment with efaroxan were investigated in the anesthetized rats. The changes in peripheral blood flow were recorded at 30 min after injection of allantoin. All values are presented as mean ± SEM (*n* = 8). *P < 0.05 as compared to the data in before.

The peripheral blood flow was markedly increased by allantoin (0.5 mg/kg, i.v.) after treatment for 30 min in the anesthetized rats, compared with the vehicle-treated control. However, as shown in Figure 4, this effect was also deleted by coadministration of efaroxan at effective dose (1.5 mg/kg, i.v.) [17]. This result reflects the decrease of total peripheral resistance of arterioles by allantoin via an activation of I-1R.

## 4. Discussion

In the present study, we found that allantoin induced a dose-dependent reduction of MBP in SHRs at 30 minutes later, the most effective time point. In anesthetized rats, the heart rate, mean arterial pressure, and cardiac contraction (*dP*/*dt*) were also significantly reduced by allantoin in a way blocked by efaroxan. Moreover, the peripheral blood flow was markedly increased by allantoin in these anesthetized rats. Thus, to the best of our knowledge, this is the first study to indicate that allantoin shows the central antihypertensive action in rats. Moreover, it seems likely that an activation of I-1R is required for this action of allantoin.

Imidazoline I1-receptors (I1-IRs) are known to be expressed in the rostral ventrolateral medulla (RVLM) of nucleus tract solitary (NTS) that seems essential for the sympathoinhibitory action of clonidine-, rilmenidine-, and moxonidine-like antihypertensive agents [23, 24]. These agents were introduced to reduce blood pressure mainly through an activation of specific receptors in RVLM [23, 24]. It has been documented that I1- IR agonist(s) may provide more useful therapy of hypertension than clonidine due to the low incidence of the side effect(s) including sedation [25].

Imidazoline receptors (I-Rs) have been introduced to play a role in the regulation of cardiovascular function [26]. The antihypertensive agent rilmenidine lowered the blood pressure via an activation of central I1-IR leading to the peripheral sympathoinhibition [27]. Antihypertensive drugs through lowering of central sympathetic nervous system (SNS) activity may contribute to reducing the heart rate, cardiac contractility, and vascular tone leading to a marked decrease of blood pressure [28, 29]. In the present study, intravenous injection of allantoin relieves the blood pressure through the decrease of cardiac output and peripheral resistance. These results suggested that the effects of allantoin is mainly through its' action in the brain. Also, heart rate, mean arterial pressure, and cardiac contractility (*dP*/*dt*) were significantly reduced in anesthetized rats by allantoin in a way blocked by efaroxan. The antihypertensive action of allantoin through an activation of I1-IR in brain can thus be elucidated.

It is generally recognized that both human and experimental hypertension are mainly characterized by the higher intravascular pressure due to constriction of vascular smooth muscle cells (VSMCs) in arteries, and this behavior, known as myogenic tone, is a key element of hypertension [30, 31]. Vascular tone is an important factor in the regulation of blood pressure [32]. Although blood pressure is regulated by multiple factors, it is generally agreed that the level of blood pressure, and particularly in hypertension, is determined in large part by total peripheral resistance that is primarily a main function of the resistance of terminal arterioles [33]. Then, we detected the peripheral arterial flow to reflect the total peripheral resistance. The peripheral blood flow was markedly increased by allantoin in anesthetized rats. Moreover, this action of allantoin was deleted by coadministration of efaroxan at the dose sufficient to block I1-IR. Then, decrease of total peripheral resistance by allantoin via activation of I1-IR can be identified. The central antihypertensive effect of allantoin can thus be confirmed.

Allantoin is nature-identical, safe, and nontoxic [34]. The present study characterizes that allantoin aids in the regulation of blood pressure in animals. Allantoin is easily degraded in the intestinal tract [19] and lost the activity after oral administration [20, 21]. Antihypertensive agents are usually administrated to patients by oral intake. However, allantoin is not suitable to follow this way. Thus, modification of chemical structure to help allantoin from degradation may develop the application of allantoin in the future.

Allantoin has been mentioned to improve lipid metabolism in high fat diet- (HFD-) fed mice [35] by decreasing energy intake and epididymal white adipose tissue (eWAT) accumulation [17]. Also, allantoin may improve glucose utilization in STZ-diabetic rats [18]. In the current study, we found that allantoin produced antihypertension at the dose the same as that for antihyperglycemic action and others. Thus, allantoin seems suitable to develop as an agent for metabolic syndrome in the future.

## 5. Conclusion

According to the obtained data, we suggest that allantoin may act as central antihypertensive agent through activation of imidazoline I-1 receptor for decrease of mean arterial pressure, heart rate, and cardiac contractility. Also, increase of the peripheral blood flow by allantoin shows the lowering of total peripheral resistance in rats. Thus, allantoin has the potential to develop as a new central antihypertensive agent in the future.
